# Association of C2, a derivative of the radial artery pressure waveform, with new onset of type 2 diabetes mellitus: the MESA study

**DOI:** 10.1186/s12933-019-0868-3

**Published:** 2019-05-17

**Authors:** Daniel A. Duprez, Nkete I. Forbang, Matthew A. Allison, Carmen A. Peralta, Steven Shea, David R. Jacobs

**Affiliations:** 1 Cardiovascular Division, Department of Medicine, University of Minnesota, 420 Delaware St SE, MMC 508, Minneapolis, MN 55455 USA; 2 Department of Family Medicine and Public Health, School of Medicine, University of California, San Diego, La Jolla, CA USA; 3 Veterans Affairs San Diego Healthcare System, La Jolla, CA USA; 4 VA Medical Center, University of San Francisco, San Francisco, CA USA; 5 Department of Medicine, Vagelos College of Physicians and Surgeons and Department of Epidemiology, Mailman School of Public Health, Columbia University, New York, NY USA; 6 Division of Epidemiology and Community Health School of Public Health, University of Minnesota, Minneapolis, MN USA

**Keywords:** Arterial pressure waveform, C2, Incident diabetes type 2, Cohort study, Cardiovascular risk factors

## Abstract

**Background:**

Although microvascular dysfunction is known to result from diabetes, it might also lead to diabetes. Lower values of C2, a derivative of the radial artery pressure waveform, indicate microvascular dysfunction and predict hypertension and cardiovascular disease (CVD). We studied the association of C2 with incident diabetes in subjects free of overt CVD.

**Methods:**

Among multi-ethnic participants (n = 5214), aged 45–84 years with no diabetes, C2 was derived from the radial artery pressure waveform. Incident diabetes (N = 651) was diagnosed as new fasting glucose ≥ 126 mg/dL or antidiabetic medicine over ~ 10 years. The relative incidence density (RID) for incident diabetes per standard deviation (SD) of C2 was studied during ~ 10 years follow-up using four levels of adjustment.

**Results:**

Mean C2 at baseline was 4.58 ± 2.85 mL/mmHg × 100. The RID for incident diabetes per SD of C2 was 0.90 (95% CI 0.82–0.99, P = 0.03). After adjustment for demographics plus body size, the corresponding RID was 0.81 (95% CI 0.73–0.89, P < 0.0001); body mass index (BMI) was the dominant covariate here. After adjustment for demographics plus cardiovascular risk factors, the RID was 0.98 (95% CI 0.89, 1.07, P = 0.63). After adjustment for all the parameters in the previous models, the RID was 0.87 (95% CI 0.78, 0.96, P = 0.006).

**Conclusions:**

In a multi-ethnic sample free of overt CVD and diabetes at baseline, C2 predicted incident diabetes after adjustment for demographics, BMI and CVD risk factors. Differences in arterial blood pressure wave morphology may indicate a long-term risk trajectory for diabetes, independently of body size and the classical risk factors.

## Background

A large part of the pathogenesis in diabetes has been focused on insulin resistance and inflammation. Microvascular disease resulting from diabetes has been described as leading to diabetic retinopathy, nephropathy, and cardiomyopathy. For example Kannenkeril et al. [1] found microvascular changes in the retinal, renal, and systemic circulation in type 2 diabetes patients. Tuttolomundo et al. [2] similar found changes in arterial stiffness in diabetic foot syndrome. Benitez-Aguirre et al. [3] found that studied greater arterial stiffness was associated with later microvascular complications in type 1 diabetes patients. During the last years it has been hypothesized that microvascular dysfunction might also be a precursor of development of diabetes, independently of impaired fasting glucose [4, 5]. Microvascular dysfunction may occur as a consequence of endothelial dysfunction. Biomarkers of endothelial dysfunction were associated with a greater risk for diabetes [6–13]. A few studies demonstrated a higher incidence of type 2 diabetes with smaller baseline retinal arteriolar diameter and arteriolo–venular ratio (AVR), while venular diameters were not significantly associated with incident diabetes [14–17]. In addition, an attenuated endothelium-dependent vasoreactivity response was significantly associated with incident diabetes in patients with hypertension [18].

Measurement of arterial stiffness is of scientific interest because it is a phenotype that is an early marker for cardiovascular disease and is simple to implement clinically. It could also predict new onset diabetes, but the latter is still to be proven. During the last 2 decades, a derivative of the continuous radial artery blood pressure waveform, C2 which has been designated as either distal arterial compliance or small artery elasticity has been studied in the Multi-Ethnic Study of Atherosclerosis (MESA) [19–21]. In MESA, C2 predicted new onset of arterial hypertension, cardiovascular disease (myocardial infarction, coronary artery disease, stroke, heart failure, peripheral artery disease) and decline in renal function beyond the classical risk factors and inflammatory parameters [19–21]. There is evidence that C2 may be considered as a marker for endothelial function/microvascular function [22]. Therefore we hypothesized that C2 may be a predictor for future development of diabetes.

## Methods

### Study cohort

The MESA is a multi-center, prospective cohort designed to investigate prevalence, correlates, and progression of subclinical (asymptomatic) atherosclerosis and their associations with incident clinical events. A detailed description of the study design, recruitment methods, and examination components and data collections has been published [23]. In brief, participants included 6814 men and women (age 45–84) of Caucasian, Hispanic, African–American and Chinese descent, free from clinically manifest CVD at baseline. Signed informed consent was obtained for all participants, and institutional review board approval was obtained for all participating institutions. We included 5214 participants free of diabetes and who had a C2 measurement at baseline and any follow-up visit at which a diagnosis of diabetes could be made.

### C2 measurement

At the baseline visit between 2000 and 2002, arterial waveforms were recorded for the entire cohort using the HDI/PulseWave CR-2000 (Hypertension Diagnostics, Inc., Eagan, Minnesota). Details of this procedure have previously been published [24]. In brief, a solid state pressure transducer array (tonometer) was placed over the radial artery of the dominant arm to record the pulse contour. C2 is estimated by the device from the waveform modeled as a sinusoidal function dampened by a decaying exponential. An index estimated directly from the waveform is divided by systemic vascular resistance (SVR) to obtain C2. SVR is estimated as mean arterial blood pressure/cardiac output, and cardiac output is estimated from ejection time taken from the pulse waveform, heart rate, age, height. Higher C2 is considered to represent a more favorable cardiovascular status.

### Risk factor assessment

Participants were given standardized questionnaires at baseline, which were used to obtain information on demographics, medical history, and smoking history. A medication inventory was also performed, and medications were grouped based on use to treat high blood pressure, or elevated blood glucose. Height and weight were assessed using a standardized protocol and body mass index (BMI) was defined (kg/m^2^). Blood pressure was measured three times in the seated position with an automated oscillometric sphygmomanometer after at least 5 min of rest. The average of the last 2 measurements was used. Standard measurements were taken for height and weight, and venous blood samples were obtained after a 12 h fast for measurements of total cholesterol, high-density lipoprotein (HDL) cholesterol, triglycerides and glucose. Physical activity (min/week in sports and dance) and TV watching (min/week) was assessed by a questionnaire.

### Study endpoint: diabetes mellitus type 2

Among those free of diabetes at baseline according to self-reported clinical statement, baseline fasting plasma glucose < 126 mg/dL and no use of anti-diabetic medications at baseline, incident diabetes was defined as fasting plasma glucose ≥ 126 mg/dL or start of anti-diabetic medications during the consecutive MESA follow-up visits. Note that it was not possible to distinguish incident type 1 from type 2 diabetes, but at MESA ages most diabetes would be type 2. Diabetes was identifiable only at clinic visits, which occurred at mean 1.64 years after baseline in 97.5% of participants, 3.20 years in 93.5%, 4.84 years in 91.3% and 9.47 years in 75.6% of participants.

### Statistical analysis

Descriptive statistics for the study cohort were summarized by means (SD) and ranges for continuous variables, and frequencies for categorical variables following five C2 categories with equal intervals, but open-ended extremes (Table 1). Over a median follow-up of approximately 10 years, we computed the relative incidence density for new onset diabetes per standard deviation of C2 as a continuous variable and separately for each C2 category using Poisson regression using 4 levels of adjustment: model 1 adjusted for age, race, sex; model 2 based on model 1 with further adjustment for body size (BMI and height; note that although height is part of the mathematical definition of BMI, BMI and height are uncorrelated); model 3 based on model 1 with further adjustment for risk factors (heart rate, blood pressure and antihypertensive therapy, blood lipids and cholesterol lowering therapy, smoking, and physical activity); and model 4 including all of the above variables. The analysis of C2 categories was also represented graphically. The study participants with borderline diabetes are at much higher risk for incident diabetes; this may differentially influence diabetes risk by C2 status. We therefore also examined the interaction with baseline impaired fasting glucose status and C2 in prediction of new onset diabetes. Because C2 correlates with BMI, we calculated the Pearson correlations (sex-adjusted) with BMI at baseline. In a subset analysis, we also calculated the Pearson correlations (sex-adjusted) with different contributing parameters to BMI, which were visceral fat (VAT), abdominal intermuscular adipose tissue (IMAT) and muscle lean volumes (LEAN). The latter measurements were obtained in a subset of participants by computed tomography at MESA exams 2 or 3 (means 1.64 and 3.20 years after MESA baseline, respectively) [25].

**Table 1:** Participant characteristics following five C2 categories (with equal intervals with open-ended extremes) in the Multi-Ethnic Study of Atherosclerosis, 2000–2002

	C2 categories (mL/mmHg × 100)									
	0.81 to < 2 (N = 797)		2 to < 4 (N = 1977)		4 to <6 (N = 1156)		6 to < 8 (N = 633)		8 to 20.74 (N = 651)	
	Mean	SD	Mean	SD	Mean	SD	Mean	SD	Mean	SD
C2 (mL/mmHg × 100)	1.64	0.26	2.90	0.58	4.91	0.57	6.91	0.58	10.44	2.06
Age (years)	68.1	9.1	64.0	9.7	59.2	9.3	57.0	9.2	54.1	7.9
White (column %)	33		40.2		43.3		45.7		50.8	
Chinese	16.9		11.2		10.7		10.7		10.3	
Black	25.5		27.9		25.2		24.8		17.4	
Hispanic	24.6		20.7		20.8		18.8		21.5	
Male	30.9		39.7		50.2		59.9		72.2	
Height (cm)	161.2	8.6	164.8	9.4	167.9	9.4	170.2	9.4	173.4	9.2
HR (b/min)	63.4	9.9	62.4	9.5	63.0	9.2	61.7	8.3	60.7	8.1
SBP (mmHg)	138.3	22.3	129.0	21.0	121.8	18.1	115.8	16.2	112.7	14.1
DBP (mmHg)	74.1	10.7	72.1	10.4	71.9	10.1	70.3	9.6	70.5	9.6
HTN Rx (%)	40.9		33.7		27.7		19.1		14.3	
BMI (kg/m^2^)	26.5	4.7	27.9	5.1	28.7	5.6	28.3	5.7	28.3	5.1
Former smoker (%)	31.2		36		37.6		36.8		39.6	
Current smoker	12.5		13.8		12.2		11.5		10.9	
Glucose (mg/dL)	91.1	10.7	89.6	10.3	89.7	10.7	88.2	10.6	88.1	9.8
Chol. (mg/dL)	197.1	35.0	196.8	35.3	194.0	34.2	193.3	34.7	190.1	33.7
HDL-C (mg/dL)	54.5	15.6	52.7	15.0	50.7	14.3	50.2	14.8	48.0	13.5
Trig. (mg/dL)	129.4	68.0	128.1	76.6	127.6	73.5	122.7	74.6	127.1	80.6
Chol. Rx (%)	19.3		15.2		14.4		12.3		8.9	
Sport and dance phys. activity (min/week)	319.4	1069.2	351.5	964.4	341.5	806.8	432.6	1094.8	507.3	1179.6
TV watching (min/week)	932.6	635.9	903.2	639.4	842.6	613.0	789.9	638.2	721.9	540.1

*HR* heart rate, *SBP* systolic blood pressure, *DBP* diastolic blood pressure, *HTN Rx* antihypertensive therapy, *BMI* body mass index, *Chol* cholesterol, *HDL-chol* high-density cholesterol, *Trig.* triglycerides, *Chol. Rx* cholesterol lowering therapy, *Phys. Activity* physical activity, *TV watching* television watching

We considered P < 0.05 to be noteworthy in general screening of findings. Analyses were performed using PC-SAS, version 9.4 (SAS Institute, Cary, NC).

## Results

Table 1 summarizes the characteristics of the study participants following C2 categories. Mean C2 at baseline was 4.58 ± 2.85 mL/mm Hg × 100. C2 was lower with a higher age. Females had lower C2 than males. Height was associated with higher C2. Participants with a lower C2 had higher systolic and diastolic blood pressure and they received more antihypertensive therapy. Participants with lower C2 had a more sedentary lifestyle. Study participants with a higher C2 tended to have higher BMI.

**Fig. 1:**
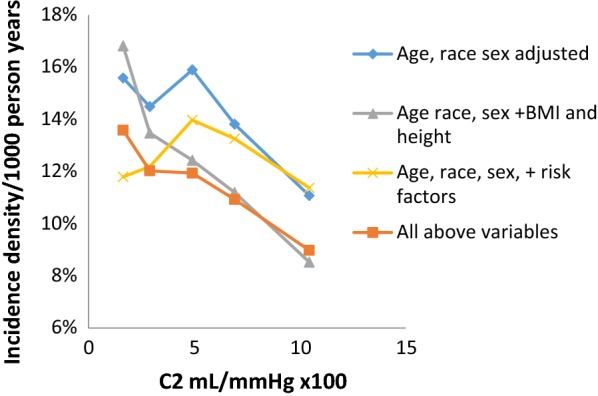
Incident diabetes by categories of C2 at 4 levels of adjustment. Adjustment for body size (BMI and height) accentuated the association of C2 with diabetes, while adjustment for cardiovascular risk factors attenuated the association. *BMI* body mass index, risk factors: heart rate, blood pressure and antihypertensive therapy, blood lipids and cholesterol lowering therapy, smoking, physical activity

Incident DM occurred in 651 participants during approximately 10 years follow-up. In Table 2, after adjustment for demographics, the relative incidence density (RID) for incident DM per standard deviation of C2 was 0.90 (95% CI 0.82–0.99, P = 0.03). After additional adjustment beyond demographics for body size (namely, BMI and height) the corresponding RID was 0.81 (95% CI 0.73–0.89, P < 0.0001); BMI was the dominant covariate in model 2. Omission of height from the model had little effect on the solution (data not shown). After adjustment for cardiovascular risk factors beyond demographics, the RID for incidence of DM for C2 was 0.98 (95% CI 0.89, 1.07, P = 0.63). In model 4 in which there was an adjustment for demographics, BMI, height and CV risk factors the RID for DM was 0.87 (95% CI 0.78, 0.96, P = 0.0.006). Figure 1 illustrates the incidence of DM by categories of C2. Whereas adjustment for CV risk factors tended to attenuate the association of C2 with incident diabetes (model 3), the strongest inverse association of C2 with incident diabetes occurred in model 2, adjusting for demographics, BMI and height; the association in model 2 is accentuated compared to the one in model 1 with adjustment for demographics alone. There was no significant interaction between impaired fasting glucose status and C2 in prediction of incident diabetes (P = 0.99). No race/ethnicity interaction with C2 was observed (data not shown).

**Table 2:** Relative incidence density (RID) diabetes per standard deviation of C2 over approximately 10 years of follow-up

	Incident diabetes				P
	N at risk	RID	95% CI, lower	95% CI, upper	
Model 1	5214	0.90	0.82	0.99	0.03
Model 2	5214	0.81	0.73	0.89	< 0.0001
Model 3	5167	0.98	0.89	1.07	0.63
Model 4	5167	0.87	0.78	0.96	0.006

Model 1: adjusted for age, race, and sex; Model 2: Model 1 + BMI (body mass index) and height; Model 3: Model 1 + risk factors (heart rate, blood pressure and antihypertensive therapy, blood lipids and cholesterol lowering therapy, smoking, and physical activity); Model 4: All above variables

In a subset of 1629 participants (Table 3) in whom IMAT, LEAN, and VAT were assessed at exams 2 or 3, although C2 at exam 1 was positively correlated with BMI. C2 was positively associated with muscle LEAN assessed 2–4 years later. BMI, IMAT and VAT form a triad with pairwise correlations of 0.57–0.61, P < 0.0001, but it is of note that although IMAT and LEAN have an inverse correlation (r = − 0.21, P < 0.0001), BMI and LEAN have a positive correlation (r = 0.29, P < 0.0001).

**Table 3:** Pearson correlations (sex-adjusted) in 1629 participants among C2 and several measures of body mass index (BMI, at baseline = exam 1) and abdominal intermuscular adipose tissue (IMAT), muscle lean volume (LEAN) and visceral adipose tissue (VAT) obtained by computed tomography at MESA (mean 1.64 and 3.20 years after MESA baseline, respectively = exam 2 and 3)

	Mean ± SD		BMI	IMAT	LEAN	VAT
C2 (mL/mmHg × 100)	4.6 ± 2.9	r	0.10	− 0.04	0.28	− 0.03
		P	0.0001	0.14	<.0001	0.27
BMI (kg/m^2^)	27.8 ± 4.9	r		0.57	0.29	0.61
		P		< 0.0001	< 0.0001	< 0.0001
IMAT (cm^2^)	2.5 ± 1.3	r			− 0.21	0.60
		P			< 0.0001	< 0.0001
Muscle LEAN (cm^2^)	13.4 ± 3.8	r				0.08
		P				0.002
VAT (cm^2^)	159.7 ± 82.1	r				1
		P				

Although no participants had diagnosed diabetes at baseline (exam 1), some participants had a diagnosis of diabetes by the time of the CT assessment

## Discussion

In a multi-ethnic sample free of overt CVD and diabetes at baseline, a higher C2 predicted significantly lower incident diabetes after adjustment for demographics, BMI and CVD risk factors. As such, changes in arterial blood pressure wave morphology may manifest a long-term risk trajectory for diabetes independently of demographics, body size and the classical risk factors. This finding is in agreement with a finding in the Malmö Diet and Cancer cardiovascular cohort, for another measure of arterial stiffness, increased carotid-femoral pulse wave velocity, which was associated with increased incidence of diabetes independent of other risk factors [26].

Among variables based on the pulse waveform, only C2 has been shown to be measure of the competence of the microcirculation [22]. Pulse wave velocity, which depends only on the arrival time of the waveform, is most studied. The assumption that connects pulse wave velocity to arterial stiffness is that a stiffer tube carries a pressure wave at higher velocity than a flexible tube. However, smaller arteries are not considered in this assumption. Other measures do depend on the shape of the waveform. Reflection magnitude [27] is based on the idea that when a forward pressure wave travels through a stiff artery, a reflection wave is formed, and pressure reflects back towards the origin of the forward wave. The augmentation index is computed after applying a transfer function to approximate the central waveform. The augmentation index is the ratio of the second to the first peak in the central waveform. The second peak is thought to be higher in the presence of a reflection wave.

The measures C1 and C2 were initially derived from a third order Windkessel model of compliance in an electrical circuit. The model assumes two capacitors, and when the capacitances C1 * R and C2 * R are divided by the systemic resistance, R, two systemic compliance measures, C1 and C2, result. The link to arterial stiffness is less direct in this case then in the case of the previously discussed measures. The proprietary owners of C1 and C2 explored the solution to the Windkessel model and suggest that C1 strongly reflects the rapid decay of pressure during diastole, while C2 is a measure of the dampening oscillations as the waveform proceeds from late systole until mitral valve closure during diastole. The dampening of these oscillations could be the result of a superimposed reflection wave. C2 also has a microcirculatory dimension. Gilani et al. [22] measured C1 and C2 in 10 healthy volunteers (3 male and 7 female) before and after inhibiting dilation of the small vessels by intra-arterial infusion of the substituted arginine NG-nitro-l-arginine-methyl ester (l-NAME). C2 was decreased after inhibition, while C1 was not. The designation small artery elasticity for C2, which has been used by the proprietary owners of C2 and by us, may therefore be too specific. We prefer to use the more generic name C2, recognizing that C2 uniquely among waveform interpretations has been shown to relate to the microcirculation.

In our analyses, the association of C2 and incident diabetes is dominated among covariates by the association of C2 with BMI, in which those with more favorable C2 had a higher BMI (BMI is therefore a negative confounder for the association of C2 and incident diabetes) [28]. Although the physiologic basis for the positive association of C2 with BMI is not well understood, the situation may be complex [29]. Forbang et al. further discussed this anti-intuitive finding and confirmed that C2 was positively associated with BMI, but also that C2 was inversely related to visceral adipose tissue once muscle mass was considered [25]. Clearly, BMI does not discriminate well between peripheral and harmful fat. As shown in Table 3 of the present paper, this association with BMI may have as much to do with BMI as an indicator of lean muscle volume as with BMI as an indicator of body fatness. In any case, BMI is a factor which is well known to be associated with higher incidence of diabetes, and its paradoxical association with better C2 must be considered in evaluating the association of C2 with diabetes.

Given the known association of C2 with microvascular function [22], the association we found between a low C2 and incident diabetes could have resulted from pre-existing microvascular changes. While microvascular dysfunction may occur independently of dysglycemia, we acknowledge that the microvascular dysfunction represented by low C2 could be the result of dysglycemia even below the fasting glucose cutpoint for diabetes, although in our data baseline C2 and fasting glucose were only weakly correlated. Muris et al. found in their meta-analysis that microvascular dysfunction (assessed using a variety of modalities) was associated with 10 to 49% higher incidence of type 2 diabetes [5]. This finding is consonant with the 50% higher diabetes incidence density, comparing the lowest (least favorable) to highest (most favorable) C2 categories in Fig. 1 in our study.

Strength of our study includes a representation of multiple ethnicities, both genders and a relatively large sample size. Prevalent CVD at baseline was an exclusion criterion. Our study also has the limitation that diabetes could only be diagnosed at 4 time points over the approximate 10 year follow-up period, and some late-appearing diabetes diagnoses were missed, when diabetes onset was after the last attended clinic visit. Selective survival bias is also a possibility. The observational association between C2 and incidence DM cannot prove a causal relationship and residual confounding is a possibility. A possible advantage to use the radial artery waveform for the calculation of C2 is that the arterial waveform analysis can also be used by applying mathematical algorithms to calculate cardiac output non-invasively. A study by Maddali et al. [30] used radial and femoral artery waveforms to calculate cardiac output; this measure correlated well with echocardiographic cardiac output measurements.

## Conclusions

Our study demonstrated that C2, which is a derivative from the arterial blood pressure wave morphology, predicts new onset of diabetes independently from the classical risk factors. C2 provides information about microvascular function and therefore these findings add important information in the pathogenesis of diabetes that microvascular dysfunction is an important contributor for new onset of type 2 diabetes. Future studies are warranted to examine the role of new classes of anti-diabetic drugs, which a have a beneficial effect on the microcirculation and small vessels, to prevent new development of type 2 diabetes.

## Data Availability

Data and materials are available upon request.

## Abbreviations

AVR: arteriolo–venular ratio
BMI: body mass index
C2: derivative of the radial artery pressure waveform
CI: confidence interval
CVD: cardiovascular disease
DM: diabetes mellitus
IMAT: abdominal intermuscular adipose tissue
LEAN: muscle lean volumes
l-NAME: arginine NG-nitro-l-arginine-methyl ester
MESA: Multi-Ethnic Study of Atherosclerosis
RID: relative incidence density
SD: standard deviation
VAT: visceral fat
